# Impact of Vaccination on *Haemophilus influenzae* Type b Carriage in Healthy Children Less Than 5 Years of Age in an Urban Population in Nepal

**DOI:** 10.1093/infdis/jiab072

**Published:** 2021-09-01

**Authors:** Sonu Shrestha, Lisa K Stockdale, Madhav C Gautam, Meeru Gurung, Shuo Feng, Pratistha Maskey, Simon Kerridge, Sarah Kelly, Merryn Voysey, Bhishma Pokhrel, Piyush Rajbhandari, Stephen Thorson, Bibek Khadka, Ganesh Shah, Karin S Scherer, Dominic Kelly, David R Murdoch, Shrijana Shrestha, Andrew J Pollard

**Affiliations:** 1 Oxford Vaccine Group, Department of Paediatrics, University of Oxford and the National Institute for Health Research Oxford Biomedical Research Centre, Oxford, United Kingdom; 2 Pediatric Research Unit, Patan Academy of Health Sciences, Kathmandu, Nepal; 3 Microbiology Unit, Department of Pathology and Laboratory Medicine, Patan Academy of Health Sciences, Kathmandu, Nepal; 4 Department of Pathology and Biomedical Science, University of Otago, Christchurch, New Zealand

**Keywords:** Hib, *Haemophilus influenza*, carriage, colonization, children, Nepal, vaccination

## Abstract

**Background:**

Reduction in detection of asymptomatic carriage of *Haemophilus influenzae* type b (Hib) can be used to assess vaccine impact. In Nepal, routine vaccination against Hib in children at 6, 10, and 14 weeks of age was introduced in 2009. Before vaccine introduction, Hib carriage was estimated at 5.0% among children aged <13 years in Nepal, with higher rates among children under 5. Large-scale evaluation of Hib carriage in children has not been investigated since the introduction of the pentavalent diphtheria-tetanus-pertussis/Hib/hepatitis B (DTP-Hib-HepB) vaccine in Nepal.

**Methods:**

A total of 666 oropharyngeal swabs were collected between August and December 2018 from healthy children between 6 months and 5 years of age attending the vaccination clinic at Patan Hospital, Kathmandu, Nepal. Of these 666 swabs, 528 (79.3%) were tested for Hib by culture. Demographic and vaccination data were collected.

**Results:**

Among 528 swabs tested for Hib, 100% came from fully vaccinated children. No swabs were positive for Hib (95% confidence interval, .0–.7). The absence of Hib in 2018 suggests vaccine-induced protection against Hib carriage 9 years after vaccine introduction.

**Conclusions:**

Following 3 doses of pentavalent DTP-Hib-HepB vaccine, Hib carriage in children under the age of 5 years in Nepal is no longer common. Ongoing high coverage with Hib vaccine in early childhood is expected to maintain protection against Hib disease in Nepal.

An exclusively human commensal and pathogen, *Haemophilus influenzae* is isolated predominantly from the upper respiratory tract of humans, and the species can be divided into 2 groups depending on the presence of a polysaccharide capsule. Unencapsulated strains are nonreactive with diagnostic antisera and therefore given the group name “nontypeable.” Six serotypes (a–f) are identified based on their reactive and antigenically dissimilar polysaccharide capsules [1], with *H. influenzae* type b (Hib) being the predominant cause of human disease in the absence of a Hib vaccination program.

Following colonization of the nasopharynx, Hib isolates can cause meningitis, pneumonia, and epiglottitis [2] and, by invasion of the bloodstream, can cause subsequent spread to secondary sites (collectively these sequelae are referred to as Hib disease) [3]. Before widespread Hib vaccine introduction, 371 000 deaths and >8 million cases of serious disease were attributed to Hib annually, primarily among children in resource-poor countries [3, 4]. A systematic review of studies in South Asia found that between 1990 and 2017, 13% of cases of bacterial meningitis in children <5 years of age were caused by Hib [5]. Modeling studies using publicly available disease burden measures in the era of conjugate vaccines (between 2000 and 2015) estimate that Hib-related deaths have declined by 90% globally since vaccine introduction [6].

The current Hib vaccine is comprised of Hib surface polysaccharide antigen (polyribosylribitol phosphate [PRP]) conjugated to a protein carrier, and has been recommended by the World Health Organization since 2006 [7]. Initial uncertainty surrounding the relative importance of Hib disease in Southeast Asia delayed the adoption of Hib vaccine in countries such as Nepal. The vaccine was introduced as a 3-dose pentavalent vaccine (with diphtheria, tetanus, pertussis [DTP] and hepatitis B [HepB]) at 6, 10, and 14 weeks (https://www.mohp.gov.np/eng/program/child-health-services/nip) with support from Gavi, the Vaccine Alliance in 2009 [2]. Data collected since 2011 estimate the average Hib vaccine coverage rates in infants (<1 year of age) to be 91%, and consistently above 87% [8]. The importance of a booster dose to maintain protective antibody persistence has been demonstrated in countries like the United Kingdom (UK) where resurgence of Hib cases was documented after 10 years following vaccine introduction [9, 10]; however, a booster dose has not been implemented in Nepal or most other low- and middle-income countries.

Prior to inclusion of Hib vaccination in routine immunization in Nepal in 2009, Hib carriage rates in children were estimated to be 5.0% overall and 6.4% among children between the ages of 1 and 4 years [2]. In this study, we evaluated the impact of Hib vaccine on Hib carriage rates in children <5 years old, 9 years after introduction of Hib vaccine in routine immunization in Nepal. As a historical control, we used a subset of data from a prevaccination study conducted by Williams et al [2]. We compared data from our study (hereafter referred to as Hib2018) with a subset of data (from an appropriate age range and hospital setting) conducted prior to vaccine introduction in Kathmandu in 2007 (hereafter referred to as Hib2007) [2].

## MATERIALS AND METHODS

### Study Setting, Samples, and Inclusion Criteria

Nepal is a landlocked South Asian country with population of 28 087 871 in 2018 according to the World Bank. This study was conducted between May and December 2018 at Patan Hospital, Kathmandu, Nepal, one of the pediatric referral units in Kathmandu. It provides outpatient and inpatient services, including an outpatient vaccination clinic for children aged <14 years.

Samples were collected from children at the hospital vaccination clinic and processed at the Patan Hospital microbiology laboratory. Ten percent of samples were tested for quality control at Oxford Vaccine Group, University of Oxford, UK.

After obtaining written informed consent from the parents or guardians, healthy children who were >6 months and <5 years of age were included in the study.

Children were excluded from the study if they were admitted to the hospital as inpatients, if they displayed an axillary temperature of ≥38°C or respiratory symptoms, or if they had taken antibiotics within the preceding 28 days.

### Consent and Ethical Approval

The study received ethical approval from research ethics committees in Nepal and Oxford (National Health Research Council reference: 116/2018; Oxford Tropical Research Ethics Committee reference: 10–18). With the help of auxiliary health workers, research officers approached the children’s parents or guardians and obtained written informed consent.

### Measurement Variables

Data on age, sex, family size, medical history, age of other children living at home, vaccination history, and history of antibiotic use in the preceding 28 days were collected using a standardized questionnaire by a research officer at enrollment.

### Specimen Collection and Handling

As was performed for the Hib2007 study, the back of the throat was swabbed by trained members of the research team with a cotton-tipped wooden swab stick. One oropharyngeal swab was taken for each participant. Skim milk tryptone glucose glycerine (STGG) media was prepared in the microbiology laboratory under sterile conditions and aliquots were stored at 2°C–6°C. The media was brought to room temperature 30 minutes prior to use. Oropharyngeal swabs from each participant were used to inoculate 1 mL of STGG media. Samples were stored at 2°C–6°C for 2, and no more than 4, hours before being transferred to the microbiology laboratory.

### Sample Processing

Five microliters of the STGG-swab sample was plated onto Hib antiserum agar plates supplemented by nicotinamide adenine dinucleotide (NAD), hemin, bacitracin, and sheep anti-Hib serum (National Institute for Biological Standards and Control, UK) using disposable sterile flexiloop (Himedia Laboratories, India). In addition, the swab itself was used around the periphery of the plate. The remaining STGG media was stored at –80°C. Inoculated plates were incubated at 37°C in a carbon dioxide (CO_2_) incubator with 5% CO_2_ and checked after 24 hours of incubation for iridescence and after 48 hours for precipitation in the agar surrounding a colony (resulting from binding of the Hib antiserum to the type b capsular polysaccharide, which is shed from the bacterial surface). Plates were stored for a further 5 days at 2°C–6°C. Plates were compared with positive control plates containing Hib strain 10211 from American Type Culture Collection. Plates without visible precipitation were discarded.

### *Haemophilus influenzae* Detection

Detection was performed in the same way as for the Hib2007 study: Hib colonies were identified by iridescence and/or precipitation in the agar surrounding a colony. Presence of Hib was confirmed by performing the X (heme) & V (NAD) factors dependence test. Isolates that were dependent on X and V factor were tested against polyvalent and type-specific antiserum (Difco, BD Biosciences) using latex ImmuLex *H. influenzae* agglutination test (Statens Serum Institut).

### Sample Size Calculation

A subset of data on similarly aged individuals included in the Hib2007 [2] was used to calculate the sample size needed to detect a 50% reduction in carriage. Forty-nine children with confirmed Hib carriage by latex agglutination, identified among 975 children aged 6–59 months (carriage prevalence of 5.03%), were used as historical controls to assess the impact of Hib conjugate vaccine on Hib carriage prevalence in the current study (Hib2018). A 50% reduction in carriage prevalence was expected from 5.0% to 2.5%. With a total of 700 children, the study would have 80.6% power to detect such a decrease in carriage prevalence using a 1-sided 5% significance level.

### Data Analysis

Demographic data (age, sex, number of adults in household, number of other children in household), Hib vaccination history, confirmed Hib carriage, and laboratory results were compared between Hib2018 and Hib2007 studies. Comparisons were made using χ ^2^ tests or 2-sided Fisher exact test where appropriate. The prevalence of Hib carriage among different age groups and overall for both studies was calculated with 95% confidence intervals (CIs), using binomial exact interval estimations [11]. Analyses were done using R version 3.6.1.

## RESULTS

### Recruitment

**Figure 1.**
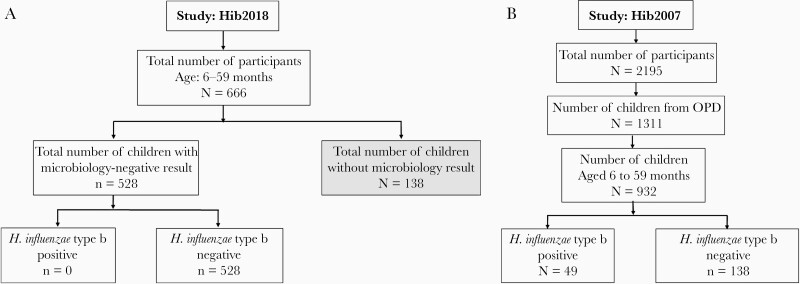
*A*, *Haemophilusinfluenzae* type b 2018 study (Hib2018; postvaccination). Altogether, 666 oropharyngeal swabs from participants aged between 6 and 59 months were taken. Of 666 samples, 528 samples were microbiologically tested. The remaining 138 samples were not tested microbiologically due to lack of reagents. The data in the gray-shaded box were removed during analysis. *B*, *Haemophilus influenzae* type b 2007 outpatient department (OPD) study (Hib2007; prevaccination) [2]. A total of 932 participants aged between 6 and 59 months were included.

Seven hundred children were to be included in the study: 267 children aged between 6 months and 11 months and 433 aged between 12 months and 59 months. A shortage of Hib antiserum meant that recruitment was halted after 666 children were enrolled. As a result of the lack of reagents, 528 (79.3%) oropharyngeal swabs were tested for Hib. Since the primary aim of the study was to find the carriage rate of Hib, other *H. influenzae* types were not tested (Figure 1A). Numbers of individuals from the Hib2007 carriage study used as the prevaccination comparator are detailed in Figure 1B.

In the Hib2007 study, children were recruited between May 2007 and December 2007, with the highest recruitment in June and the lowest in September. In the Hib2018 study, children were recruited between August 2018 and December 2018, with the highest recruitment in September (Supplementary Figure 1). Nepal has 2 respiratory seasons: from January to March and July to September.

### Demographic Data

The proportion of children in the younger age group (6–11 months) was lower in the Hib2018 study (127/528 [24.05%]) compared with the Hib2007 study (325/932 [34.87%], *P* < .001; Table 1). Despite this, the overall median age was 1.40 years for Hib2007 and 1.09 years for Hib2018. The proportion of female and male participants in each study was not significantly different (*P* = .568).

**Table 1.** Demographic Data for the Hib2007 and Hib2018 Studies

Characteristic	Hib2007	Hib2018	*P* Value
Age			
6–11 mo (±29 d)	325 (34.87)	127 (24.05)	<.001
12–59 mo (±29 d)	607 (65.13)	401 (75.94)	(18.61; *df* = 1)
Total (all ages)	932	528	
Median age, y (range)	1.40 (0.60–5.00)	1.09 (0.51–4.96)	
Sex			
Female	443 (47.53)	242 (45.83)	.568
Male	489 (52.47)	286 (54.17)	(.32; *df* = 1)
No. of adults at home			
None	1 (0.10)	0 (0)	
1–2	479 (51.39)	272 (51.52)	.078
3**–**5	287 (30.79)	187 (35.42)	(8.396; *df* = 4)
6–9	145 (15.56)	64 (12.12)	
≥10	20 (2.15)	5 (0.95)	
No. of other children <15 y old at home			
None	418 (44.85)	287 (54.36)	.009
1–2	444 (47.64)	208 (39.39)	
3–5	63 (6.76)	31 (5.87)	
6–9	4 (0.43)	2 (0.38)	
≥10	2 (0.21)	0	
Missing	1 (0.10)	0	
Hib vaccination received			
Yes	6 (0.64)	528 (100)	<.001
No	922 (98.93)	0	
Reported as unknown	4 (0.43)	0	
Hib vaccination dose			
1 dose	3 (0.32)	0	<.001
2 doses	0	0	
3 doses	2 (0.21)	528 (100)	
Reported as unknown	1 (0.11)	0	
*Haemophilus influenzae* detected			
Hib positive	49 (5.26)	0	<.001
Hib negative	883 (94.74)	528 (100)	

Data are presented as No. (%) unless otherwise indicated.

Abbreviations: *df*, degrees of freedom; Hib, *Haemophilus influenzae* type b.

Family sizes were comparable between the 2 studies. Fifty-one percent of children (479/932) included in the Hib2007 study lived with 1 or 2 adults, which is comparable with the proportion in the Hib2018 study (51.52% [272/528], *P* = .078). Similarly, 44.85% (418/932) of children recruited in the Hib2007 study lived with no other children, compared with 54.36% (287/528) of children in the Hib2018 study (Table 1).

Data included from the Hib2007 study were stratified into different groups based on the Hib agglutination test and compared with the Hib2018 study group (Table 2).

**Table 2.** Distribution of Demographic Characteristics for the Hib2007 and Hib2018 Studies

Characteristic	Hib2007^a^		Hib2018
	Hib Positive	Hib Negative	Hib Negative
Age			
6–11 mo (±29 d)	16 (32.65)	309 (34.99)	127 (24.05)
12–59 mo (±29 d)	33 (67.34)	574 (65.01)	401 (75.94)
Total (all ages)	49	883	528
Median age, y (range)	1.40 (0.7–4.6)	1.40 (0.6–5.0)	1.09 (0.50–4.96)
Sex			
Female	30 (61.22)	413 (46.77)	242 (45.83)
Male	19 (38.77)	470 (53.23)	286 (54.16)
No. of adults at home			
None	0	1 (0.11)	0 (0)
1–2	23 (46.93)	456 (51.64)	272 (51.52)
3–5	19 (38.77)	268 (30.35)	187 (35.42)
6–9	7 (14.28)	138 (15.63)	64 (12.12)
≥10	0	20 (2.27)	5 (0.95)
No. of other children <15 y old at home			
None	20 (40.81)	398 (45.07)	287 (54.35)
1–2	27 (55.10)	417 (47.23)	208 (39.39)
3–5	2 (4.08)	61 (6.91)	31 (5.87)
6–9	0	4 (0.45)	2 (0.37)
≥10	0	2 (0.23)	0
Missing	0	1 (0.11)	0
Hib vaccination received			
Yes	0	6 (0.68)	528 (100)
No	49 (100)	873 (98.87)	0
Reported as unknown	0	4 (0.45)	0
Hib vaccination dose			
1 dose	0	3 (0.34)	0
2 doses	0	0 (0)	0
3 doses	0	2 (0.23)	528 (100)
Not reported	3 (6.12)	3 (0.34)	0

Data are presented as No. (%) unless otherwise indicated.

Abbreviation: Hib, *Haemophilus influenzae* type b.

^a^Hib2007 study stratified by agglutination result.

### Hib Detection and Prevalence

Table 3 summarizes the Hib carriage rate in the Hib2007 and Hib2018 studies. In the Hib2007 study, the overall prevalence of Hib carriage in the age group studied was 5.26% (95% CI, 3.91%–6.89%). In the Hib2018 study, the prevalence was 0.00% (95% CI, .0%–.7%) (Table 3).

**Table 3.** *Haemophilus influenzae* Carriage Rate in the Hib2007 and Hib2018 Studies, by Age Group

Study	Hib Carriage Rate		
	No. of Children	No. Hib Positive	Hib Positive, % (95% Binomial Exact CI)
Hib2007^a^			
Overall	932	49	5.26 (3.91–6.89)
6–11 mo	325 (34.9)	16	4.92 (2.84–7.87)
12–59 mo	607 (65.1)	33	5.44 (3.77–7.55)
Hib2018			
Overall	528	0	0.00 (.00–.70)
6–11 mo	127 (24.1)	0	0.00 (.00–2.86)
12–59 mo	401 (75.9)	0	0.00 (.00–.92)

Abbreviations: CI, confidence interval; Hib, *Haemophilus influenzae* type b.

^a^Hib2007 study used a subset of data from appropriately aged children in the outpatient department of Patan Hospital, Kathmandu, from Williams et al [2].

The analysis of age-specific rates showed a higher incidence of Hib carriage in the 12- to 59-month age group (5.44% [95% CI, 3.77%–7.55%) compared with the 6- to 11-month age group (4.92% [95% CI, 2.84%–7.87%]) in the Hib2007 study; however, this was not statistically significant.

## DISCUSSION

In this study, we show that the prevalence of Hib carriage has declined among children aged between 6 months and 5 years since the introduction of Hib-containing DTP-Hib-HepB pentavalent vaccine in Nepal, to the point where Hib is no longer detected in this age group.

The decline of Hib carriage from a prevaccine estimate of 5% in 2007 (Hib2007) to 0% (Hib2018) in oropharyngeal swabs from the same area of Nepal shows that progress has been made toward elimination of Hib circulation in children. Similar findings have been reported in Gambian [9], Brazilian [12], and American-Indian children [13] following vaccine introduction. The study used here as a prevaccination reference (Hib2007, [2]) subsequently also provided Hib vaccine to a subset of 109 children aged 3 months to 5 years living within children’s homes for socially disadvantaged children. Having reswabbed children in these orphanages, it was found that carriage had dropped from a prevaccine prevalence of 8.9% (21/237) to 1.8% (2/109) 6 months after vaccination [2].

A carriage study conducted in 2011, 2 years after Nepal introduced the pentavalent Hib-containing vaccine for infants aged 6, 10, and 14 weeks, investigated 102 unvaccinated students aged 5–14 years in Pokhara, Nepal [14]. The study found only 2 of 102 (1.9%) swabs positive for *H. influenzae* (Hib and non-Hib *H. influenzae* were not differentiated). The students in this study may have benefitted from herd immunity whereby vaccine-induced reduction of bacterial carriage results in protection in those not immunized. While older children rarely get Hib disease and therefore may be naturally immune, comparing these low carriage rates seen in 2011 with data from older children aged 5–12.9 years within the Hib2007 study by Williams et al [2], where rates of Hib carriage were 5.0% among 813 older children, points toward some effect of herd immunity. Although nonvaccinated children were not studied here, it would be interesting to understand if the consistently high Hib conjugate vaccine coverage seen in Nepal translates into reduced carriage in nonvaccinated individuals and adults over time.

Both natural exposure to Hib and administration of Hib conjugate vaccine elicit robust anti-PRP antibodies. A threshold of anti-PRP antibody required for short-term protection has been established as >0.15 µg/mL, and long-term protection at >1 µg/mL [15]. Two studies have assessed anti-PRP antibody levels in Nepal to evaluate disease susceptibility. Marshall et al found that prior to introduction of Hib conjugate vaccine in Nepal, 80% of children under the age of 5 years had anti-PRP levels below the 0.15 µg/mL protective threshold, but only 17% of people aged between 15 and 54 years of age had nonprotective levels (highlighting the important role of natural exposure), and levels declined in individuals >55 years of age [16]. Metz et al found that all 74 vaccinated infants recruited at Patan Hospital (Kathmandu, Nepal) had levels of anti-PRP antibodies >1 µg/mL at 18 weeks after vaccination, but this dropped to 73% at 1 year after Hib conjugate vaccine [15]. Four weeks following a further booster dose at 1 year showed that 98.5% of children had surpassed the long-term protective 1 µg/mL level.

A reemergence of Hib disease cases in countries including the UK [17] and The Gambia [18] raised the possibility of waning immunity in populations vaccinated in infancy without a booster. This was seen in the UK among children aged 4 years after they had received the Hib conjugate vaccine in infancy [19] and was associated with a rise in Hib disease in all ages in 1999. The finding led to the introduction of a booster dose of Hib conjugate vaccine in a nationwide catch-up campaign, targeting children between 1 and 4 years of age, followed by use of a routine booster at 12 months of age. In an evaluation of a single booster dose it was shown that anti-PRP antibody levels increased in a group of 386 children to above the 1 µg/mL threshold 2 years later [20]. In the absence of a booster dose, evidence of Hib carriage among school-aged children in the UK remained [21]. In environments with low natural boosting such as the UK, the continued presence of Hib carriage in nonboosted children may be driving disease among adults with nonprotective levels of anti-PRP antibodies. More research is needed to understand if a booster dose is as beneficial in areas with higher natural exposure.

The magnitude and quality of anti-PRP antibodies is known to be affected by the way in which the Hib conjugate vaccine is administered. Concomitant administration of Hib vaccine with acellular pertussis vaccine has been shown to result in lower levels of anti-PRP antibodies [22] and reduced avidity maturation of those antibodies [23]. This, however, does not translate into reduced functional capacity of those antibodies in analyses in vitro [23], nor has postmarketing vaccine effectiveness of acellular pertussis–containing Hib combination vaccines (in Germany) been compromised [24]. Because whole-cell pertussis vaccine is used in Nepal, we would not expect to see this issue in the current study. Evidence also exists for decreased levels of anti-PRP antibodies with the coadministration of another conjugate vaccine, meningococcal C (MenC) [25]. While the current Expanded Programme on Immunization schedule in Nepal does not include MenC, the pneumococcal conjugate vaccine (PCV) is recommended at 6 weeks, 10 weeks, and 9 months. There is evidence to suggest that anti-Hib immunoglobulin G concentrations can be enhanced when PCV conjugated to the same carrier protein (tetanus toxoid) as used in the Hib vaccine is coadministered with Hib-containing pentavalent vaccine [26]. Close surveillance will be needed as more combinations of vaccines, and conjugate vaccines, are included.

Risk factors associated with invasive disease due to Hib include low socioeconomic status and overcrowding [27, 28]. The number of people living in a household was similar between the Hib2007 and Hib2018 studies; however, socioeconomic status was not captured. The generally accepted measure of standard of living, gross domestic product per capita, in Nepal has increased 2.8 times between 2007 and 2018 [29]. Assuming that the risk factors associated with invasive disease due to non-Hib serotypes are the same as Hib, it would be of interest to understand if this more affluent yet similarly crowded population is more or less at risk of non-Hib disease.

In both Hib2007 and Hib2018, antibiotic use in the last 28 days was an exclusion criterion. A high rate of prehospital antibiotic usage has been reported in Nepal [2], which may result in an underestimation of Hib carriage in studies with less stringent exclusion criteria. The method of plating used between the 2 studies compared here is slightly different; however, we do not see any issues with comparability between studies. In Hib2007, Williams et al used direct plating from the swab for samples collected in the outpatient department (which is used here as the comparator group) but used *Haemophilus* transport media prior to plating for samples collected in schools and homes [2]. Authors did not report any issues with comparability of techniques. For Hib2018, the swab was stored for an average of 2 hours in STGG transport media before both media and swab were used to inoculate Hib antiserum agar plates. STGG has been shown to be an excellent transport and storage media, preserving *H. influenzae* isolates for up to 3 years [30]. In that study conducted in Finland [30], carriage rates of *S. pneumoniae* and *H. influenzae* were similar to those in another study in Finland, where direct plating was used [31]. For nasopharyngeal swabs taken for *S. pneumoniae* measurement, STGG was found to enhance long-term storage without any loss in Colony Forming Units [32].

### Limitations

Since this study (Hib2018) was designed to investigate the carriage of Hib only, samples were not investigated for non-Hib *H. influenzae* serotypes. It is likely that invasive disease due to non-Hib serotypes in Nepal will follow the global trend of slow serotype replacement that has been observed following introduction of Hib vaccines in Europe [33, 34], Canada [35], and the United States [28]. Since no nonvaccinated children were included in this study, we were unable to estimate Hib carriage in unvaccinated or incompletely vaccinated children. Additionally, due to the age limits in this study, we are unable to estimate carriage in children over the age of 5 years. The 100% vaccination coverage with 3 doses here may not be representative of every child in Nepal; however, estimated Hib vaccine coverage rates in infants <1 year of age is 91%, and consistently above 87% [8].

Evidence of the seasonality of Hib carriage in Nepal is lacking; however, studies of *H. influenzae* carriage from China indicate a propensity for higher rates in winter months [36] (winter months in Nepal being November–February) and in Kenya, carriage of *H. influenzae* was found to be more prevalent during the rainy season (odds ratio, 1.8 [37]). It is unlikely that the minor differences in timing of sampling between Hib2007 and Hib2018 were a major source of bias in this study.

In conclusion, this study adds to the literature that vaccination with Hib-containing vaccines is associated with a reduction in Hib carriage in Nepal. Further studies are required to understand if what is seen in this urban setting is recapitulated in a rural setting, if serotype replacement with nontypeable and non-Hib encapsulated serotypes occurs, and if waning immunity (assisted by potential seasonal increases in carriage rates) might necessitate implementation of booster doses in order to maintain protective levels of anti-PRP antibodies on a population level.

## Supplementary Data

Supplementary materials are available at *The Journal of Infectious Diseases* online. Consisting of data provided by the authors to benefit the reader, the posted materials are not copyedited and are the sole responsibility of the authors, so questions or comments should be addressed to the corresponding author.

jiab072_suppl_Supplementary_Figure_1

## Notes

***Acknowledgments.*** The authors thank the clinical and nonclinical staff at Patan hospital, Kathmandu, Nepal. Sincere thanks to all infants and parents/guardians for allowing participation in this study. Thanks to E. J. Williams for sharing data associated with the Hib2007 study.

***Supplement sponsorship.*** This supplement is sponsored by the World Health Organization and the U. S. Centers for Disease Control and Prevention.

***Financial support.*** This project was funded by the Oxford Vaccine Group (www.ovg.ox.ac.uk) with support from the NIHR Oxford Biomedical Research Centre (http://oxfordbrc.nihr.ac.uk) and the WHO, as part of pediatric inpatient invasive bacterial disease surveillance in Nepal.

***Potential conflicts of interest.*** The authors have read the journal’s policy and have the following conflicts: A. J. P. is Chair of the DHSC JCVI and is a member of the WHO’s Strategic Advisory Group of Experts on Immunization. A. J. P. is an NIHR Senior Investigator. All other authors report no potential conflicts of interest.
