# Supplementary material for: Impact of Vaccination on Haemophilus influenzae Type b Carriage in Healthy Children Less Than 5 Years of Age in an Urban Population in Nepal
Source: J Infect Dis. 2021 Sep 1;224(Suppl 3):S267–74. doi: 10.1093/infdis/jiab072 (PMC8409530; doi:10.1093/infdis/jiab072)
Supplement: jiab072_suppl_Supplementary_Figure_1 [file jiab072_suppl_supplementary_figure_1.docx]

**Supplementary Figure 1:** Number of participants recruited by calendar month for Hib2007 (top graph) and Hib2018 (bottom graph)
